# Population Genetic Structure of the Dwarf Seahorse (*Hippocampus zosterae*) in Florida

**DOI:** 10.1371/journal.pone.0132308

**Published:** 2015-07-22

**Authors:** Nathan Fedrizzi, Melanie L. J. Stiassny, J. T. Boehm, Eric R. Dougherty, George Amato, Martin Mendez

**Affiliations:** 1 Department of Ecology, Evolution and Environmental Biology, Columbia University, New York, New York, United States of America; 2 Sackler Institute for Comparative Genomics, American Museum of Natural History, New York, New York, United States of America; 3 Department of Ichthyology, Division of Vertebrate Zoology, American Museum of Natural History, New York, New York, United States of America; 4 Department of Ecology, Evolution and Behavior, CUNY Graduate Center, New York, New York, United States of America; 5 Department of Environmental Science, Policy and Management, University of California, Berkeley, California, United States of America; 6 Latin America and Caribbean Program, Wildlife Conservation Society, Bronx, New York, United States of America; University of Innsbruck, AUSTRIA

## Abstract

The dwarf seahorse (*Hippocampus zosterae*) is widely distributed throughout near-shore habitats of the Gulf of Mexico and is of commercial significance in Florida, where it is harvested for the aquarium and curio trades. Despite its regional importance, the genetic structure of dwarf seahorse populations remains largely unknown. As an aid to ongoing conservation efforts, we employed three commonly applied mtDNA markers (ND4, DLoop and CO1) to investigate the genetic structuring of *H*. *zosterae* in Florida using samples collected throughout its range in the state. A total of 1450 bp provided sufficient resolution to delineate four populations of dwarf seahorses, as indicated by significant fixation indices. Despite an overall significant population structure, we observed evidence of interbreeding between individuals from geographically distant sites, supporting the hypothesis that rafting serves to maintain a degree of population connectivity. All individuals collected from Pensacola belong to a single distinct subpopulation, which is highly differentiated from the rest of Floridian dwarf seahorses sampled. Our findings highlight the utility of mtDNA markers in evaluating barriers to gene flow and identifying genetically distinct populations, which are vital to the development of comprehensive conservation strategies for exploited taxa.

## Introduction

The dwarf seahorse (*Hippocampus zosterae*) is a diminutive species endemic to North America, distributed from southern Texas throughout the northern coast of the Gulf of Mexico, including the entirety of the Florida Peninsula and the northern islands of the Bahamas [1]. *H*. *zosterae* is one of three seahorse species found in the western North Atlantic, but it is the only New World seahorse demonstrating dwarfed morphology, reaching a maximum length of only 38.0 mm [1,2]. The species was listed as Vulnerable in the IUCN Red List of Threatened Species in 1996 due to its long history of human exploitation for the aquarium and curio trades and suspected declines in both occurrence and quality of available habitat. However, in 2003 this designation was changed to Data Deficient owing to a lack of current and reliable data [3].

Dwarf seahorse habitat is characterized by shallow, near-shore sand flats ranging from one to four meters in depth, typically dominated by seagrass beds and macroalgae [1,4,5]. These habitats often occur within sheltered lagoons or embayments, with reduced exposure to strong currents and heavy wave action [6]. Such requirements render *H*. *zosterae* highly vulnerable to the effects of anthropogenic activities, such as dredging for coastal development and bottom trawling for commercial harvesting of shrimp, which share the same habitat [7,8,9]. As with many other seahorse species, the morphological adaptations that make *H*. *zosterae* efficient as a sedentary ambush predator also hinder its potential for active dispersal, promoting high site fidelity to small home ranges [10]. Limited long-range vagility, combined with patchy distributions and the requirement for sheltered coastal habitats, can result in wide separation of seahorse populations by geographic distance or hydrological features, potentially reducing the likelihood of migration and active dispersal between populations [11].

Dwarf seahorses typically produce at least three generations per year, with four or more generations possible in the southern parts of their range [1]. Most breeding activity occurs between February and October, with breeding timing and juvenile growth both closely associated with day length and regional water temperatures [1,12]. During the summer months, when water temperatures exceed 30°C, male dwarf seahorses may produce up to two broods per month, each containing up to 55 offspring. Juvenile development is rapid, with individuals reaching reproductive maturity between two and three months of age.

Dwarf seahorses are benthic at birth, settling onto the surrounding substrate and vegetation shortly after they emerge from their father’s pouch [1,13]. Adult *H*. *zosterae* are poor swimmers and may experience even more limited mobility and a greater risk of predation as a result of their small size. Long-range dispersal of *H*. *zosterae* is likely restricted to instances of passive dispersal through rafting, which can occur as vegetative holdfasts break loose from the substrate and are carried by ocean currents [4]. Genetic exchanges between non-contiguous seahorse populations are presumed to be rare and restricted to rafting events and limited pelagic dispersal [2,11]. Long distance dispersal through rafting has been documented in larger seahorse species, though the success of rafting as a dispersal strategy depends chiefly upon chance and favorable current patterns [14].

In marine environments, where barriers to dispersal are often cryptic, genetic sampling has proven a useful tool to investigate breaks in connectivity occurring between geographically close populations of benthic species [15,16,17]. Understanding genetic population structure is a valuable tool for conservation, as it allows for the identification of genetically distinct populations and the establishment of management units that can be employed to protect particularly vulnerable populations [11,18]. Such efforts are especially important in species that are commercially harvested and may therefore be vulnerable to overexploitation.

Here we assess the population structure of one such species, *Hippocampus zosterae*, in the state of Florida utilizing three mitochondrial DNA markers (ND4, DLoop and CO1) as tools for an initial assessment of genetic structuring within an exploited species.

## Methods

### Ethics statement

This study was conducted in accordance with American Veterinary Medical Association (AVMA) guidelines and all steps were taken to avoid needless suffering. The following protocol for all sampling procedures employed in this study was reviewed and specifically approved by the Columbia University Institutional Animal Care and Use Committee (IACUC) Protocol number: AC-AAAE9612 (Y1 M00) and was administered by the authors. Tricaine methane sulfonate (MS-222) was used as an anesthetic agent at a range of 25-50mg/L as suggested by the ICM veterinary consult. All field collections for this study were conducted in locations that permit public fishing, and nonresident fishing licenses for saltwater shoreline fishing issued through the Florida Fish and Wildlife Conservation Commission were obtained prior to all sampling efforts. Field collections did not involve endangered or protected species and no additional specific permissions were required for sampling procedures or experimental manipulations. Seahorses collected from Big Pine Key and Tampa Bay were collected through a partnership with licensed marine ornamental collectors and were fin-clipped while in holding tanks. Individuals collected in this manner could not be returned to the wild and were donated as breeding stock to an aquaculture facility in Ft. Pierce, Florida (Seahorse Source). Seahorses from all other locations were collected by dip netting and released after fin clipping to the site of collection.

### Sample collection

Tissue samples were collected from *H*. *zosterae* individuals from eight locations around the Florida Peninsula during August 2012 and January 2013 (Table 1). Field collections were performed through a combination of snorkeling and dip netting. Fin clips were taken from the lower corner of the dorsal fin and stored in 95% ethanol as specified by Lourie [19] and Lourie et al. [20]. Fin clips of thirteen dwarf seahorses from Indian River, Florida, were provided by a collaborating researcher who obtained them from the Florida Department of Fish and Wildlife as part of a phylogenetic study of Atlantic seahorse diversification [2].

**Table 1:** Sampling locations, site codes and genetic diversity indices.

Location	Site Code	*n*	H*n*	π (SD)	K (SD)	Tajima’s D (P)	Fu’s F (P)
Plantation Key	**A**	19	19	7.85937 (4.08528)	9.95900 (1.96550)	**-1.94083** (0.00800)	**-13.28602** (0.00000)
Tavernier	**B**	9	9	6.80289 (3.98578)	5.93800 (1.27428)	**-1.64242** (0.03700)	**-3.93205** (0.01100)
Lower Matecumbe Key	**D**	23	12	6.41407 (3.55904)	-0.06397 (0.90015)	-1.22823 (0.09200)	-0.99354 (0.33700)
Bradenton Beach	**E**	11	11	8.29993 (4.76410)	7.29000 (1.50405)	-1.19167 (0.13000)	**-4.81435** (0.00900)
Big Pine Key	**F**	19	15	6.29673 (3.54677)	9.13700 (2.06361)	-1.36253 (0.05800)	**-5.79438** (0.01300)
Tampa Bay	**G**	22	21	9.16453 (5.01921)	11.70700 (2.15030)	**-1.69514** (0.02600)	**-12.14403** (0.00000)
Indian River	**H**	6	6	6.143791 (3.427238)	4.31100 (1.02924)	-0.38669 (0.40900)	-2.03476 (0.05800)
Pensacola	**I**	18	16	6.21145 (3.37410)	8.78800 (1.90176)	**-1.92428** (0.01500)	-8.60416 (8.60416)

Table includes sample size (*n*), number of haplotypes (H*n*), mean nucleotide diversity (π), and mean number of pairwise differences among sequences (K); significant values are shown in bold.

A total of 170 seahorses were fin-clipped for this study. Site codes were assigned according to the order in which the populations were sampled (Table 1). One population (C) contained only two individuals and was therefore removed from the study. Five juvenile individuals (F27, F28, F29, F30 and F31) were removed from the dataset to avoid the possibility of including offspring birthed by one of the males during the collection process. The remaining 163 individuals were initially included in the dataset, but 36 of these were subsequently removed due to low quality sequence data or a failure to amplify all three mtDNA gene regions (ND4, DLoop, CO1), leaving a total of 127 individuals from eight sampling locations in the final dataset.

### DNA extraction and sequencing

DNA was extracted from preserved fin clips using a DNeasy Blood and Tissue Kit (Qiagen) and amplified through PCR. Amplification reagents consisted of: 22.3μl deionized water, 0.28 uM forward primer, 0.28 uM reverse primer, 301.55 mM BSA, one Illustra PuReTaq Ready-To-Go PCR bead and 1.0μl template DNA (25-50ng). The same thermocycler profile specified by Woodall [21] was used for all amplifications (denaturing for 3 minutes at 95°C, 34 cycles at 95°C for 30s, annealing at 50°C for 30s, extension for 30s at 72°C, final extension at 72°C for 3 minutes, followed by a hold at 10°C). Three primers for mitochondrial gene regions (ND4, DLoop and CO1) were created from a full mitochondrial genome of *Hippocampus comes*, downloaded from GenBank (Accession #: NC_020336) [22] (S1 Table). An optimized fast STeP cycle sequencing protocol was used for all samples, with 10μl total reaction volume per well. Sequencing reaction components included: 0.5μl Big Dye, 2.0μl extension buffer (500 mM Tris/HCl, pH 8.3, 80 mM MgCl2, and 300 mM KCl), 0.64 uM primer, 3.5μl dH_2_0 and 2.0μl PCR product [23]. Analysis of sequence reactions were completed using a 96-well HITACHI Applied Biosystems ABI 3730XL DNA Analyzer (model no. 625–0020).

### Data analysis

Forward and reverse DNA sequences were aligned and edited in *Sequencher v*. *5*.*0*.*1* (Gene Codes Corporation, Anne Arbor, MI). All contiguous sequences below 90% quality were re-sequenced; those still below 90% quality were then excluded from the analysis. Aligned sequences from all three mtDNA markers were used in a BLAST search conducted on *Geneious R6*.*06* using sequences from GenBank for reference. All CO1 sequences were estimated above 99% confidence as *H*. *zosterae*. No reference sequences existed on GenBank for ND4 and DLoop gene regions. Contiguous sequences were trimmed so that all were the same length and exported by mtDNA gene region as FASTA concatenated files. All sequences were checked for stop codons to ensure that no mitochondrial pseudogenes (NUMTs) were present in the sample. Concatenated files from all gene regions (ND4, DLoop and CO1) were combined, aligned and further trimmed in Textwrangler (Bare Bones Software, Inc.) to produce a single concatenated file with a 1450 bp consensus region. Sequences were deposited in Genbank (accession numbers: DLoop: KJ495805-KJ495951; CO1: KJ621693–KJ621831; ND4: KJ621832–KJ621982).

The diversity of mtDNA sequences was estimated at the haplotype level using *DNA Sequence Polymorphism v*. *5*.*10*.*1* (DNA SP), which assigned 104 haplotypes from the overall population of 127 individuals [24]. This haplotype assignment was supported by an identical result using the ‘haplotype collapser’ in *FaBox v*.*1*.*41* [25]. Differentiation of populations was assessed through an Analysis of Molecular Variance (AMOVA) of mtDNA haplotypes assigned in DNA SP and conducted in *Arlequin 3*.*5* [26,27]. To assess the proportion of nucleotide and allelic diversity among subpopulations in relation to that present in the overall population, we calculated pairwise Φ_ST_ and *F*_ST_ values between all sampled populations [28]. Though similar, *F*_ST_ assumes distances between alleles to be equal and responds to vicariance more quickly, while Φ_ST_ considers pairwise distances between alleles and increases more slowly after population subdivision, presenting a more historical view of population dynamics [29,30]. To assess intrapopulation genetic diversity and historical demography, we calculated nucleotide diversity (π) in *Arlequin*. We also calculated two neutrality statistics, Tajima’s D and Fu’s F, to differentiate our results from neutral variation [31,32].

To observe patterns of genetic distance and variation between identified haplotypes in the sample we used *Network v*.*4*.*611* to produce a median joining network with an epsilon value of zero [33]. Networks are more suitable than trees for visualizing the structure of genetic data at the population level, as they assume the presence of ancestral haplotypes in the population [34]. Due to high haplotype frequency, it was necessary to reduce complexity by calculating a maximum parsimony median joining network and employing a star contraction algorithm with a maximum star radius of ten [35]. To investigate patterns of isolation by distance we conducted a Mantel test of autocorrelation between geographic and genetic distances in IBD Web Service v.3.32 [36]. The significance of the Mantel test was assessed at 30,000 randomizations. To further explore patterns of differentiation between sampling sites, an assessment of DNA divergence was conducted in *DNA SP v*. *5*.*10*.*1* [24]. The number of fixed differences (FD) and shared mutations (SM) observed in pairwise comparisons were calculated, as well as Jukes-Cantor corrected variances of the average pairwise number of nucleotide differences per site (D_xy_) between all sampled sites (S1 and S2 Figs).

## Results

Table 1 illustrates the sample sizes for each of the eight sites in Florida and provides an overview of genetic data. A total of 104 maternal haplotypes were identified from the overall sample, many of which were unique to *H*. *zosterae* individuals. Haplotype assignment in *DNA SP* showed shared haplotypes to be regionally aggregated, with the same haplotype occurring only within neighboring populations. A notable exception to this distribution was observed in individuals from site F, which shared haplotypes with individuals collected from both the west coast locations (sites E, G) and the Florida Keys locations (sites A, B, D). Mean nucleotide diversity (π) and mean number of pairwise differences among sequences (K) was highest in individuals collected from the west coast of Florida (sites E, G). Tajima’s D and Fu’s F were negative for all sampled sites, and both measures were significantly negative for individuals collected from sites A, B and G (Table 1).

The Analysis of Molecular Variance (AMOVA) conducted in *Arlequin 3*.*5* was significant for molecular distance (Φ_ST_) and haplotype frequencies (*F*_ST_) in the overall sample (Φ_ST_ = 0.46909, P < 0.00000; *F*_ST_ = 0.02791, P < 0.00000). Pairwise comparisons between sampling sites demonstrated significant genetic structuring among Floridian dwarf seahorses (Table 2). Four putative populations were delineated by combining proximate sampling locations with non-significant Φ_ST_ and *F*_ST_ values, such that individuals collected from sites A, B and D were collapsed into a single Eastern Keys population (Table 3). Seahorses from the two west coast sampling locations, sites E and G, were also combined, forming a West Coast population. Pairwise comparisons between individuals from site I and those collected from all other sites yielded high and significant pairwise Φ_ST_ and *F*_ST_, supporting their demarcation as a discrete putative population (Pensacola). Seahorses from site F also demonstrated high and significant pairwise fixation index values when compared to seahorses from the majority of other sites, and were retained as the fourth discrete putative population (Big Pine Key). However, the Big Pine Key group was less statistically supported than the other identified populations, as fixation index data showed seahorses from Big Pine Key to be significantly differentiated from the Pensacola and West Coast populations, but not significantly different from all of the sampling sites included in the Eastern Keys population (Tables 2 and 3). Seahorses from site H had significant Φ_ST_ values only in pairwise comparisons with those from sites I and E, and were not delineated as a discrete population or included within any other putative population due to the small size of the sample and lack of consistency in its relationship to the other identified populations (Tables 2 and 3).

**Table 2:** Pairwise fixation indices measured between sampling locations.

	A	B	D	E	F	G	H	I
A	-	-0.02634	-0.00383	**0.05174**	0.03014	**0.04961**	0.00387	**0.75083**
B	-0.03077	-	0.00585	**0.06250**	0.04720	0.03366	0.00849	**0.77426**
D	-0.00060	0.01786	-	0.07015	**0.07868**	**0.07889**	0.05698	**0.77635**
E	**0.12379**	**0.11743**	**0.19546**	-	**0.11211**	0.00466	0.07650	**0.75336**
F	0.02804	0.02952	**0.09716**	**0.15780**	-	**0.09227**	0.03060	**0.77868**
G	**0.12526**	**0.09875**	**0.19864**	0.00348	**0.14091**	-	0.03514	**0.72775**
H	-0.00210	0.00756	0.04230	**0.16175**	0.05570	**0.13252**	-	**0.78916**
I	**0.75773**	**0.78162**	**0.78854**	**0.74817**	**0.78434**	**0.72092**	**0.80118**	-

Pairwise comparisons using three mtDNA markers in *Arlequin 3*.*5*; Φ_ST_ is shown on the upper diagonal; Jukes-Cantor corrected *F*_ST_ values are presented on the lower diagonal; significant values are shown in bold.

**Table 3:** Pairwise fixation indices measured between putative populations.

	Eastern Keys	West Coast	Big Pine Key	East Coast	Pensacola
Eastern Keys	-	**0.06339**	**0.05292**	0.02484	**0.76102**
West Coast	**0.06391**	-	**0.08820**	0.03895	**0.72454**
Big Pine Key	**0.05284**	**0.08866**	-	0.03060	**0.77868**
East Coast	0.02382	0.03802	0.02952	-	**0.78916**
Pensacola	**0.78206**	**0.74717**	**0.79807**	**0.80728**	-

Pairwise comparisons using three mtDNA gene regions in *Arlequin 3*.*5*; Φ_ST_ is shown on the upper diagonal; Jukes-Cantor corrected *F*_ST_ values are presented on the lower diagonal; significant values are shown in bold.

**Fig 1:**
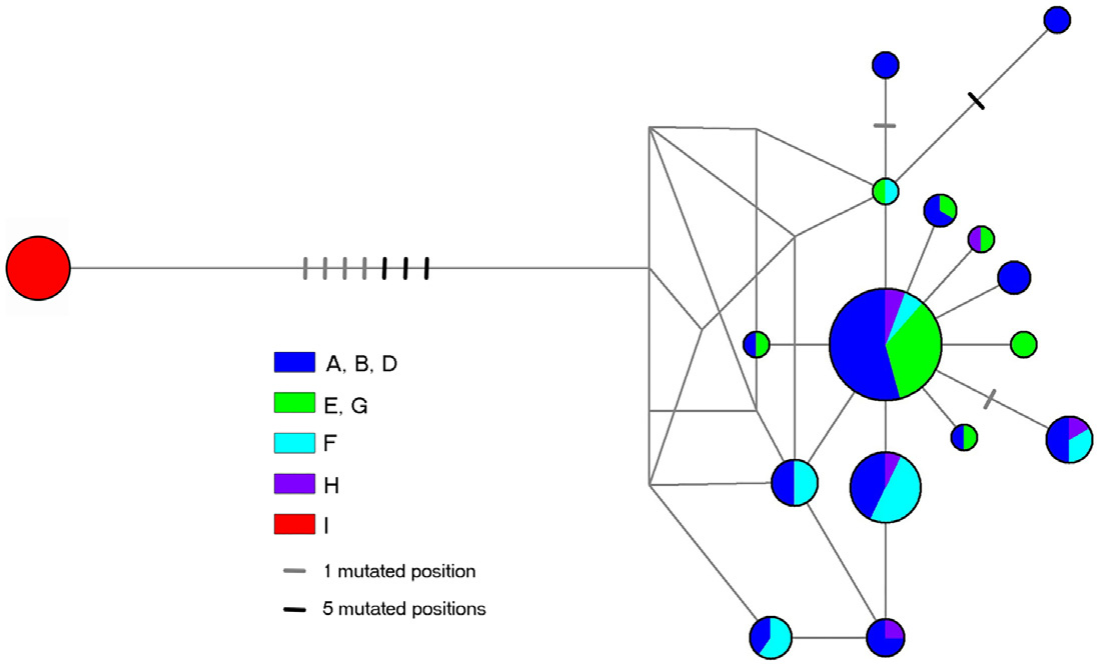
Maximum parsimony median joining Network. Network complexity was reduced with a star contraction algorithm with a maximum star radius of ten; circle size is proportionate to haplotype frequency and colors reflect groupings of populations established through non-significant pairwise Φ_ST_ and *F*_ST_ (Table 2); hatch marks represent additional mutational steps separating haplotypes; visualization produced in *Network v*.*4*.*611* (Bandelt et al. 1999).

The maximum parsimony median joining network produced in *Network v*.*4*.*611* shows that, with the exception of individuals collected from site I, haplotype groups were broadly distributed throughout the sample (Fig 1). There was some variability of haplotype frequencies within the peninsular Florida sub-network, with haplotypes from the Eastern Keys (in blue) being more commonly distributed than those from the West Coast (in green), which were more clustered. The sixteen haplotypes identified from individuals collected from Pensacola were condensed into a single node by the star contraction algorithm, and were separated from all other haplotype groups by the longest branch of the network, with nineteen missing additional mutational steps separating haplotypes (shown as hatch marks).

**Fig 2:**
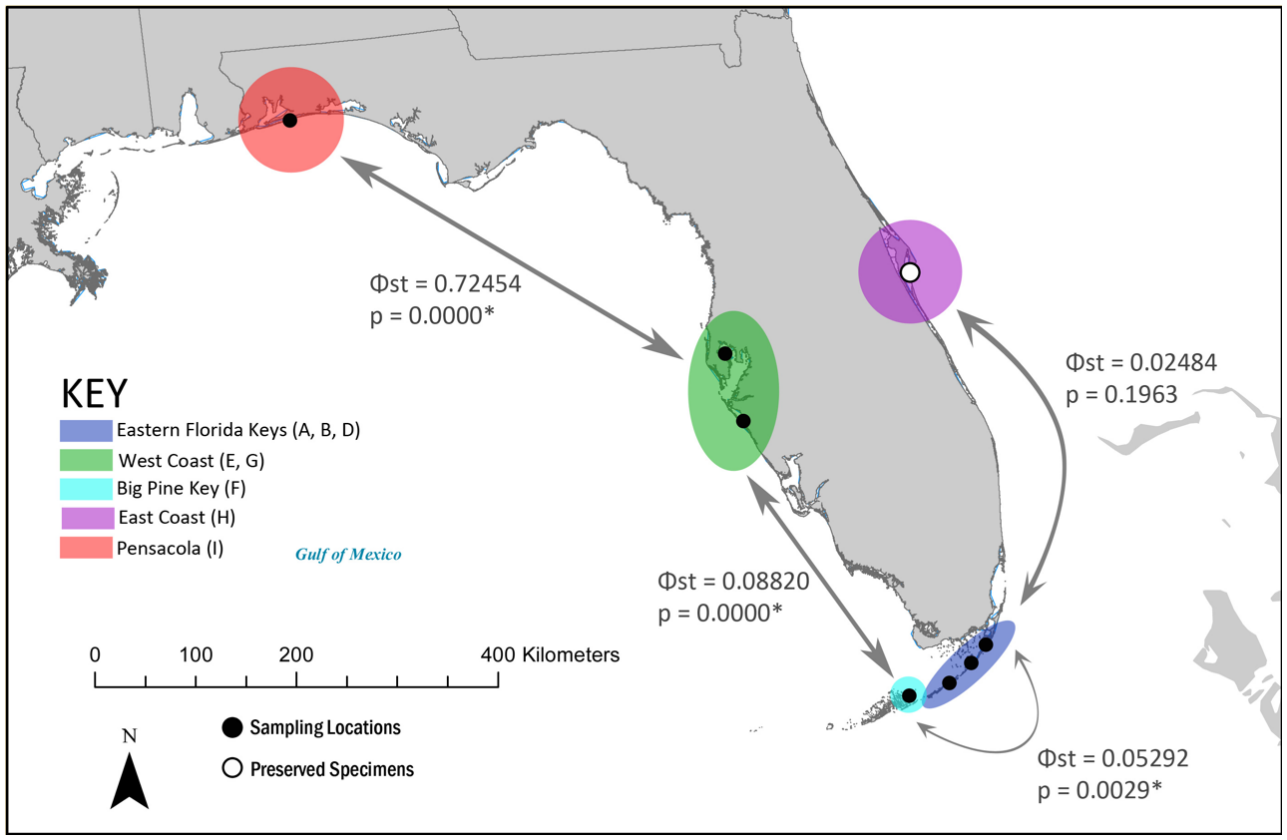
Pairwise Φ_ST_ measured between adjacent groups of sampling sites. Groups were determined through non-significant pairwise Φ_ST_ and *F*_ST_ (Tab. 2).

Pairwise Φ_ST_ values were calculated between adjacent groups, which were the most geographically proximate and assumed to have the greatest potential to exchange genes through passive dispersal (Fig 2). All comparisons of pairwise Φ_ST_ between adjacent groups were significant with the exception of those involving site H. The greatest pairwise Φ_ST_ was observed between the Pensacola and West Coast populations, with Φ_ST_ values and statistical significance remaining the same or decreasing as the comparisons move eastward along the Florida coast.

**Fig 3:**
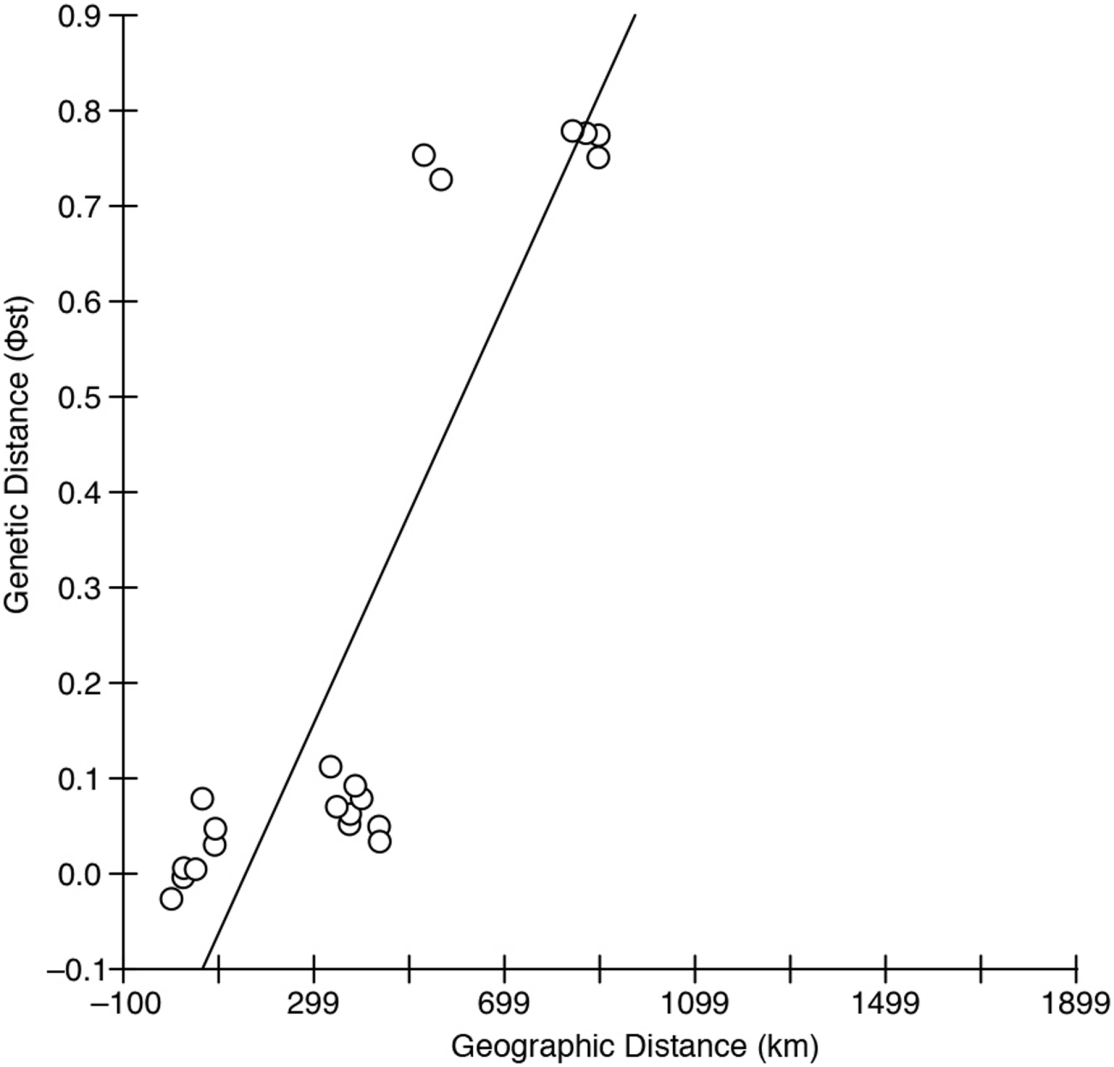
Mantel test of correlation between geographic and genetic distance. Matrix correlation between pairwise values of geographic distance (km) and genetic distance (Φ_ST_) showed a significant correlation, P = 0.0042, from 30,000 randomizations.

The Mantel test of autocorrelation between genetic and geographical distance conducted in IBD Web Service indicated a significant pattern of isolation by distance (Φ_ST:_ P = 0.0042, *F*_ST:_ P = 0.0040 from 30,000 randomizations) (Fig 3). Pairwise comparisons of genetic distance between adjacent sampling sites frequently yielded low fixation index values, and genetic distance typically increased in value and significance with increased geographic distance (Table 2). Similarly, comparisons between putative populations show a weak pattern of increasing Φ_ST_ correlated with increasing geographic distance (Table 3).

## Discussion

### Population structure

Our findings indicate significant population structuring among *Hippocampus zosterae* populations in Florida. We present strong evidence for the presence of a genetically distinct dwarf seahorse population (Pensacola), delineate two recognizable populations (Eastern Keys and West Coast), and suggest the presence of a fourth (Big Pine Key). The consistently high and strongly significant values for the various estimates of genetic differentiation employed in this study support the demarcation of the Pensacola sample as markedly genetically differentiated from the rest of the seahorses collected in this study and the consideration of the population as a discrete management unit [37]. Our analyses also lend credence to the suggestion made in previous studies that rafting may serve as a common means of passive dispersal facilitating genetic connectivity between geographically distant seahorse populations [2,4,21,38].

### Isolation by distance

Our results support an isolation by distance model as a contributing driver of population structuring in Floridian *H*. *zosterae*, in which geographically proximate populations are more genetically similar than those that are farther apart [36]. These findings implicate a stepping-stone or nearest-neighbor model in the structuring of dwarf seahorse populations, though other organismal and environmental factors are likely influential, including rafting and current patterns [39].

### Rafting

The network visualization and genetic distance map suggest that, with the possible exception of Pensacola, some gene flow occurs between sampled locations. As the distances between these locations far exceed the capacity of *H*. *zosterae* for active migration, these findings implicate passive dispersal as a common mechanism underpinning genetic connectivity in the species. Passive dispersal is likely accomplished through rafting, which has been previously documented in other seahorse species [38,40], and Woodall et al.’s [14] reported occurrence of a North American seahorse in European waters was likely the product of a long-range dispersal by rafting. As dwarf seahorses produce benthic offspring and have limited capacity for active dispersal, rafting provides a likely explanation for our findings of genetic intermixing between distant populations [1, 13].

### Gulf current patterns

Palumbi [15] implicated oceanic currents as an important factor influencing larval transport and genetic differentiation in passively dispersing marine species. As such, the population structure of a passively dispersing species in the Gulf of Mexico may reflect the influence of the surface Loop Current, a large permanent current affecting the eastern Gulf of Mexico and the Florida Peninsula [41,42]. The shape and extent of the current fluctuate seasonally and annually, but the overall pattern involves a clockwise northerly flow of water from the Caribbean Sea, which loops through the Gulf of Mexico and flows eastward through the Straits of Florida before turning north as it rounds the Florida peninsula [43].

The pattern of decreasing Φ_ST_ from west to east along the Florida coast identified through pairwise comparisons of genetic distance between contiguous sampling sites may signal migration facilitated by the Loop Current, in which the effective distance is compressed for eastwardly migrating individuals. The influence of the current may also increase the effective distance for westward migration, which would constitute what Palumbi [44] described as “an invisible barrier” that sets biogeographical boundaries by creating directional gradients that constrain gene flow via passive dispersal. Such a barrier could be a contributing factor to the significant *F*_ST_ and Φ_ST_ measured between the adjacent sampling sites D and F. The considerable distance between Pensacola and the typical northern extent of the Loop Current may prevent passively dispersing individuals from gaining access to the current, potentially contributing to the lack of genetic intermixing observed between Pensacola and all other populations [43]. However, rare intrusions of the Loop Current into coastal areas of the northern Gulf coast have been documented, suggesting that infrequent instances of facilitated southern dispersal may occur [45].

### High haplotype diversity

The ratio of haplotypes to individuals identified in this analysis could be considered uncommonly high in general terms. Goswami et al. [46] conducted mtDNA population analyses using 350bp cytochrome *b* fragments from two large *Hippocampus* species (*H*. *kuda* and *H*. *trimaculatus*) with pelagic juvenile phases and found substantially lower measures [5,47,48]. This discrepancy is likely due to their use of a single gene and the very different natural history of *H*. *zosterae* as a rapidly reproducing, short-lived species. Dwarf seahorses produce at least three generations annually throughout their range, exhibiting a seasonal boom-bust cycle of population expansion that typically peaks in September, likely coinciding with a similar rise in genetic variation [1,28]. Additionally, at least one measure of neutrality was found to be significantly negative for six of the eight sampled sites, indicating a history of widespread population expansion throughout Florida.

Differences in developmental strategy may also contribute to the variation in haplotype diversity observed among *Hippocampus* species. Species that produce pelagic offspring display prolonged periods of planktonic suspension that increase access to ocean currents and maximize capacity for long-range dispersal [49]. This may allow for regular genetic exchange between meta-populations and contribute to greater haplotypic homogeneity over evolutionary time. Species with directly benthic juveniles, such as *H*. *zosterae*, lack the ability to broadcast offspring over long distances, potentially resulting in greater retention of regional haplotypes and elevated heterozygosity within the overall population.

A similarly high level of haplotype diversity was observed by Tollis et al. [50] in their study of green anole (*Anolis carolinensis*) populations throughout the southeastern United States, which assigned 128 haplotypes to a total of 191 individuals in an analysis of concatenated 1172bp sequences from multiple mtDNA markers. Similar to *H*. *zosterae*, green anoles are widely distributed organisms with relatively short lifespans and strong potential for rapid population growth [1,50]. Greater resolution in an assessment of haplotype diversity in *H*. *zosterae* populations could be achieved by analyzing nuclear DNA, which typically yields much lower levels of haplotype diversity than observed in analyses of mitochondrial DNA [50].

### Management implications

Assessments of population structure that reveal significant genetic differentiation in the absence of obvious population breaks or subdivisions often result in the designation of new management units [18]. The recognition of management units may be particularly useful in conservation efforts for marine species, as population differentiation is often cryptic and results from the combined effects of physiological limitations and subtle environmental barriers [15]. Our findings support the designation of four management units of dwarf seahorses in Florida (Pensacola, West Coast, Eastern Keys and Big Pine Key). The Pensacola population, which was found to be genetically distinct, may be of particular interest with regards to management for the purposes of conservation.

The trade in dwarf seahorses for both the aquarium and curio trades has historically been concentrated in Florida [51,52]. Collection of *H*. *zosterae* in Florida is regulated in the same manner as other near-shore marine fishes and is subject to the restrictions of recreational and commercial fishing licenses issued by the Florida Fish and Wildlife Conservation Commission [51]. The results of our analyses, considered alongside observations of the methods of dwarf seahorse collectors, suggest that the impact of the aquarium trade on *H*. *zosterae* is likely small and can be sustained by dwarf seahorse populations. Implementation of seasonal restrictions on large-scale catches of dwarf seahorses, such as those for the curio trade, could be a useful management tool for curtailing collection during the peak of annual population contraction, which occurs from December to February [1,52].

Further study is needed to determine if the Pensacola population is truly genetically isolated or is part of a larger population spanning the northern coast of the Gulf of Mexico, including Mississippi, Alabama and the rest of the Florida panhandle. Future research incorporating both morphological and nuclear data with samples collected across the range of *H*. *zosterae* will be useful in resolving the full population dynamics of the species.

## Supporting Information

S1 Fig Pairwise fixed differences and shared mutations. DNA Divergence between sampling sites calculated in *DNA SP v*. *5*.*10*.*1*, showing the number of fixed differences (FD) and shared mutations (SM) observed in pairwise comparisons.
(TIF)

S2 Fig Average pairwise number of nucleotide differences. DNA Divergence between subpopulations implemented in *DNA SP v*. *5*.*10*.*1*, showing the number of Jukes-Cantor corrected variances of the average pairwise number (±SE) of nucleotide differences per site (D_xy_) between all sampled sites.
(TIF)

S1 Table Primers designed for this study. Sequences and properties of primer pairs for sequenced mtDNA gene regions (ND4, DLoop, and CO1).
(TIFF)

S2 Table IBD data. Background data tests conducted in IBD Web Service; pairwise estimates of geographic distance (rounded to the nearest km) between sampling locations are shown on the upper diagonal; pairwise estimates of genetic distance (Φ_ST_) are shown on the lower diagonal.
(XLSX)
