# Supplementary figures and images for: Population Genetic Structure of the Dwarf Seahorse (Hippocampus zosterae) in Florida
Source: PLoS One. 2015 Jul 22;10(7):e0132308. doi: 10.1371/journal.pone.0132308 (PMC4511636; doi:10.1371/journal.pone.0132308)

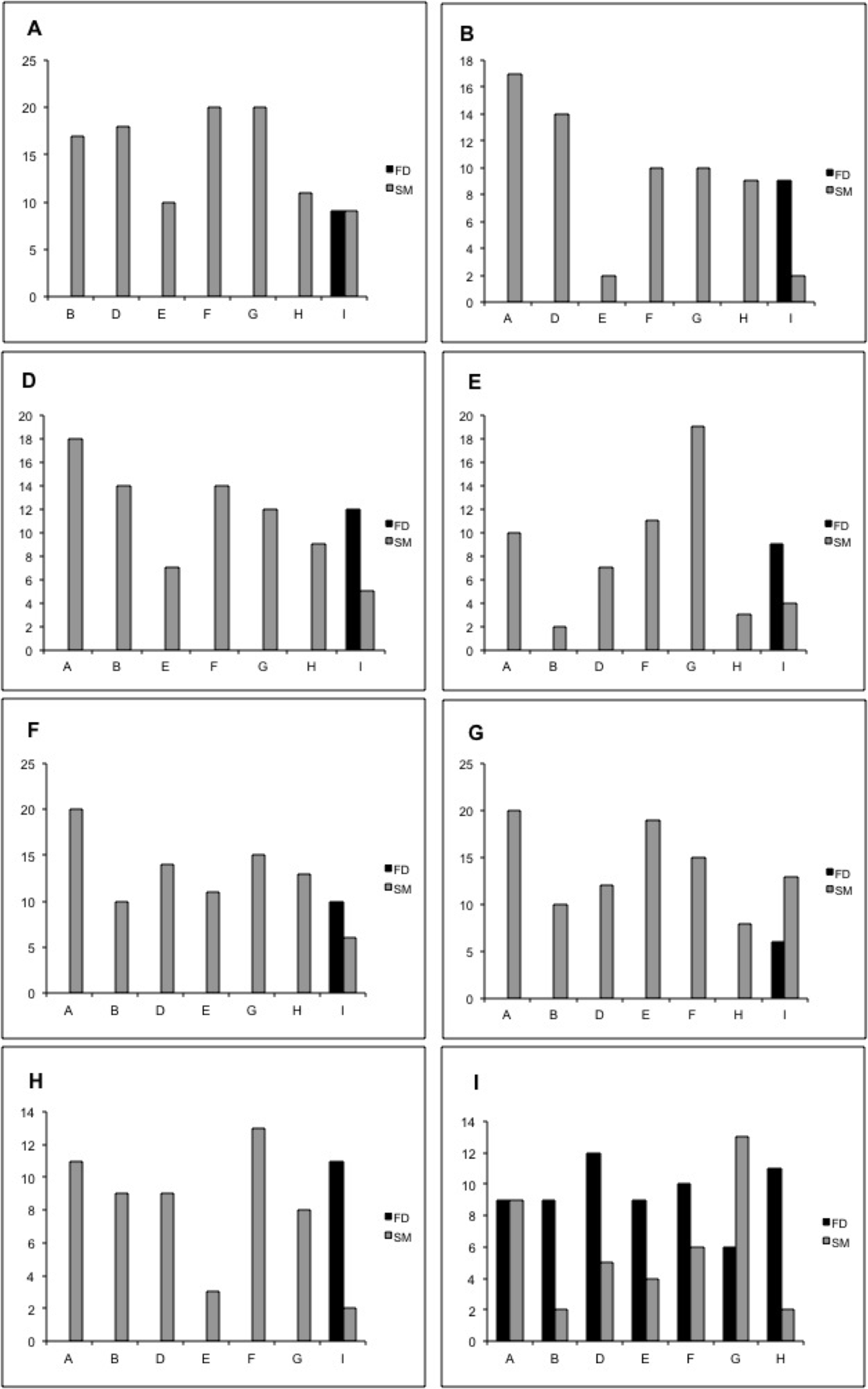

Supplement: S1 Fig — DNA Divergence between sampling sites calculated in DNA SP v. 5.10.1, showing the number of fixed differences (FD) and shared mutations (SM) observed in pairwise comparisons. (TIF) [file pone.0132308.s001.tif]

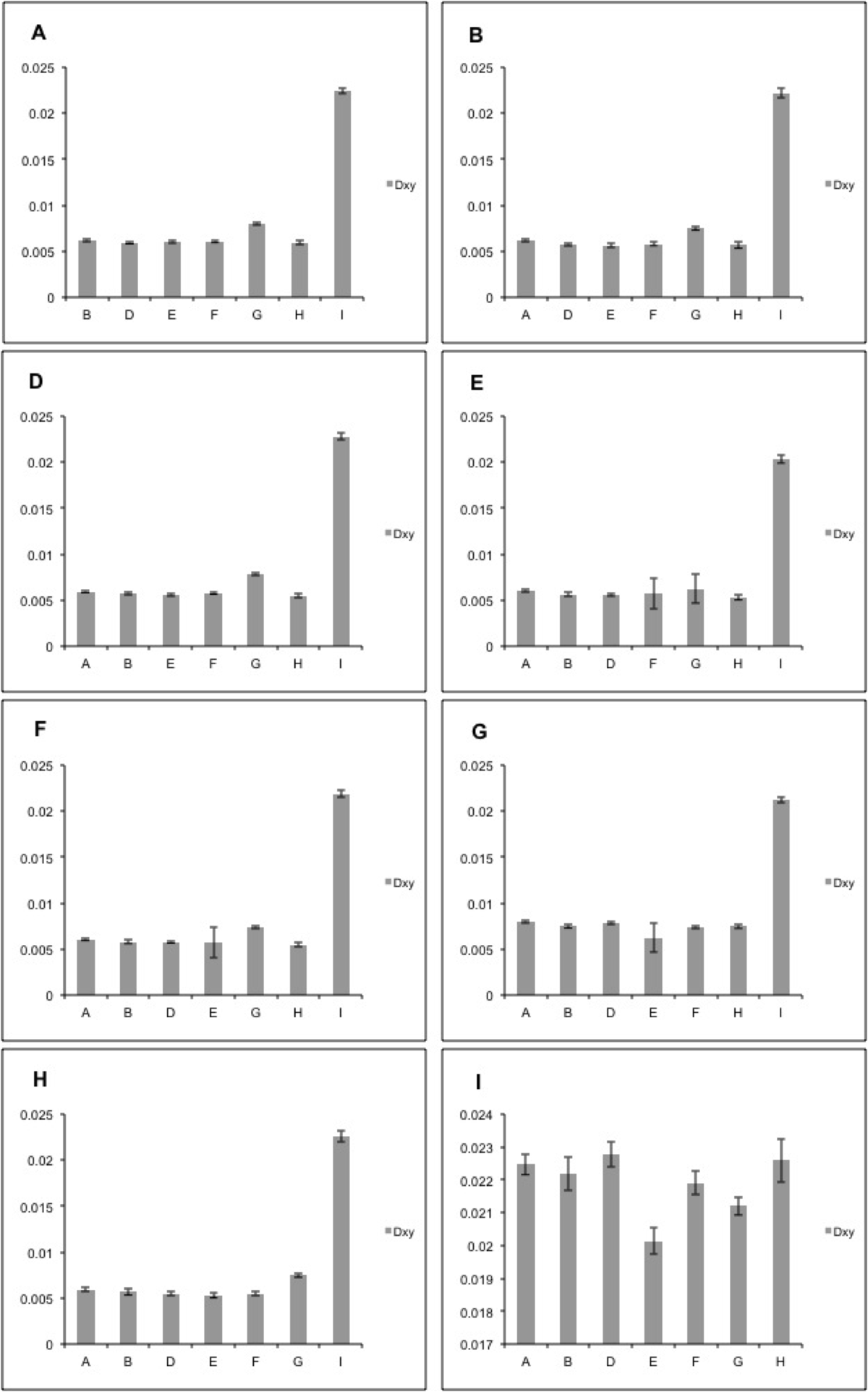

Supplement: S2 Fig — DNA Divergence between subpopulations implemented in DNA SP v. 5.10.1, showing the number of Jukes-Cantor corrected variances of the average pairwise number (±SE) of nucleotide differences per site (Dxy) between all sampled sites. (TIF) [file pone.0132308.s002.tif]

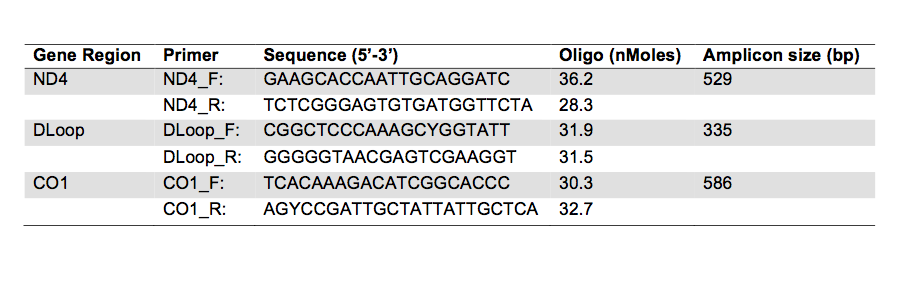

Supplement: S1 Table — Sequences and properties of primer pairs for sequenced mtDNA gene regions (ND4, DLoop, and CO1). (TIFF) [file pone.0132308.s003.tiff]
